# β-Glucans (*Saccharomyces cereviseae*) Reduce Glucose Levels and Attenuate Alveolar Bone Loss in Diabetic Rats with Periodontal Disease

**DOI:** 10.1371/journal.pone.0134742

**Published:** 2015-08-20

**Authors:** Viviam de Oliveira Silva, Raquel Vieira Lobato, Eric Francelino Andrade, Cristina Gomes de Macedo, Juliana Trindade Clemente Napimoga, Marcelo Henrique Napimoga, Michel Reis Messora, Ramiro Mendonça Murata, Luciano José Pereira

**Affiliations:** 1 Department of Veterinary Medicine, Physiology and Pharmacology Area, Federal University of Lavras, Lavras, Minas Gerais, Brazil; 2 Laboratory of Orofacial Pain, Department of Physiological Sciences, Piracicaba Dental School, University of Campinas, Piracicaba, São Paulo, Brazil; 3 Laboratory of Immunology and Molecular Biology, São Leopoldo Mandic Institute and Research Center, Campinas, São Paulo, Brazil; 4 Department of Surgery and Bucco-Maxillofacial Traumatology, Division of Periodontics, School of Dentistry of Ribeirão Preto, University of São Paulo, Ribeirão Preto, São Paulo, Brazil; 5 University of Southern California, Herman Ostrow School of Dentistry, Division of Periodontology Diagnostic Sciences, Dental Hygiene & Biomedical Science, Los Angeles, California, United States of America; 6 Department of Health Sciences, Physiology and Pharmacology Area, Federal University of Lavras, Lavras, Minas Gerais, Brazil; University of Florida, UNITED STATES

## Abstract

The objective of this study was to assess the effects of oral ingestion of β-glucans isolated from *Saccharomyces cereviseae* on the metabolic profile, expression of gingival inflammatory markers and amount of alveolar bone loss in diabetic rats with periodontal disease. Diabetes *mellitus* was induced in 48 Wistar rats by intraperitoneal injection of streptozotocin (80 mg/kg). After confirming the diabetes diagnosis, the animals were treated with β-glucans (by gavage) for 28 days. On the 14^th^ day of this period, periodontal disease was induced using a ligature protocol. β-glucans reduced the amount of alveolar bone loss in animals with periodontal disease in both the diabetic and non-diabetic groups (p < 0.05). β-glucans reduced blood glucose, cholesterol and triacylglycerol levels in diabetic animals, both with and without periodontal disease (p < 0.05). Furthermore, treatment with β-glucans reduced the expression of cyclooxygenase-2 and receptor activator of nuclear factor kappa-B ligand and increased osteoprotegerin expression in animals with diabetes and periodontal disease (p < 0.05). It was concluded that treatment with β-glucans has beneficial metabolic and periodontal effects in diabetic rats with periodontal disease.

## Introduction

Periodontal disease is characterized by chronic inflammation that affects tooth support tissues and may lead to tooth loss [1,2]. Although the primary etiologic factor for this disease is the presence of dental biofilm and the associated host response, systemic diseases such as diabetes *mellitus* modulate the development and progress of periodontal disease [3,4].

Diabetes *mellitus* is a disease in which carbohydrate, protein and lipid metabolism homeostasis is inadequately regulated by the pancreatic hormone insulin, resulting in an increase in blood glucose levels [5]. Periodontal disease is the sixth most common co-morbid condition in patients with diabetes *mellitus* [6,7] and evidence indicates a bidirectional relationship between these two pathologies [8,9]. In non-compensated diabetic patients, hyperglycemia promotes vascular alterations (microangiopathies) as well as inefficacious phagocytosis by neutrophils and macrophages. In addition, it causes retardation of collagen synthesis, which delays tissue repair [10]. The chronic release of pro-inflammatory cytokines in active periodontal disease reduces insulin action and consequently aggravates these metabolic alterations [11].

Naturally derived compounds such as β-glucans have shown promise for blood glucose and immune response control in both animal and human studies [12–14]. β-glucans are the main structural components of fungi, plants, cereal grains and some bacterial cell walls [13]. The different origins of these polysaccharides as well as their physico-chemical characteristics determine their main action [15]. β-glucans found in plants and grains (β-1,3/1,4) are known to promote metabolic activity [16], while those from fungi (β-1,3/1,6) promote immune activity [15,17].

β-glucans have shown the capacity to modulate both specific and non-specific immune responses [13], stimulating the production of pro-inflammatory cytokines and phagocytosis [18,19]. Orally administered β-(1,3/1,6) glucan solutions have been used in periodontal disease models and have reduced alveolar bone loss [12,20]. Also, these polymer fibers have known hypoglycemic action [14,21], since they form a gelatinous layer in the intestine, thereby reducing the absorption of carbohydrates [15,22,23].

The efficacy of β-glucans as an immunomodulatory agent in periodontal disease in diabetic patients is poorly understood. In addition, there is a scarcity of studies investigating the effects of these compounds on the association of these two diseases, which emphasizes the importance and novelty of this study. Because of the bidirectional relationship and significant co-morbidity between diabetes *mellitus* and periodontal disease, research involving a compound that is able to modulate inflammatory response and concomitantly reduce blood glucose levels is of extreme interest. This fact is even more important when we take into account that periodontal disease is more frequent and more severe in patients with inadequate blood glucose control [24]. Furthermore, because periodontal disease and diabetes *mellitus* show high prevalence and have chronic characteristics [10], the improvement of control techniques is relevant from the public health perspective. Thus, the present study aimed to assess the effects of ingesting β-glucans isolated from *Saccharomyces cereviseae* on the metabolic profile and alveolar bone loss parameters in diabetic rats with periodontal disease.

## Materials and Methods

### Animals

The present study was approved by the Ethics Committee for Animals Use (CEUA) from the Federal University of Lavras (UFLA) through protocol number 083/11, according to national legislation enforced by the National Council of Animal Experimentation Control (CONCEA) of Brazil.

A total of 48 healthy male adult rats (*Rattus norvegicus albinus*, *Wistar*) weighing 192 *±* 21 g (approximately seven weeks old) were used in the present study. The animals were provided from UFLA Central Bioterium (conventional bioterium). A totally causalized design was used, with a factorial scheme 2x2x2 (diabetic or not; induced to periodontal disease or not, treated or not treated with β-glucans), with n = 6 animals per group. The animals were submitted to a seven-day period of acclimation with the environment and the project execution team. They were placed in individual metabolic cages and the room was acclimatized to a 22 ± 2°C temperature, with 12/12 hour light/dark cycles. Commercial animal food and pure water were supplied *ad libitum* during the whole experimental period.

After the acclimation week, the 48 animals were randomly divided into eight groups. A power calculation was performed to determine the sample size. The animal was considered the study unit. The sample size was determined to provide 80% power to recognize a significant difference of 20% among groups and a standard deviation of 15% with a 95% confidence interval (α = 0.05). Therefore, a sample size of six animals per group was required.

**Fig 1:**
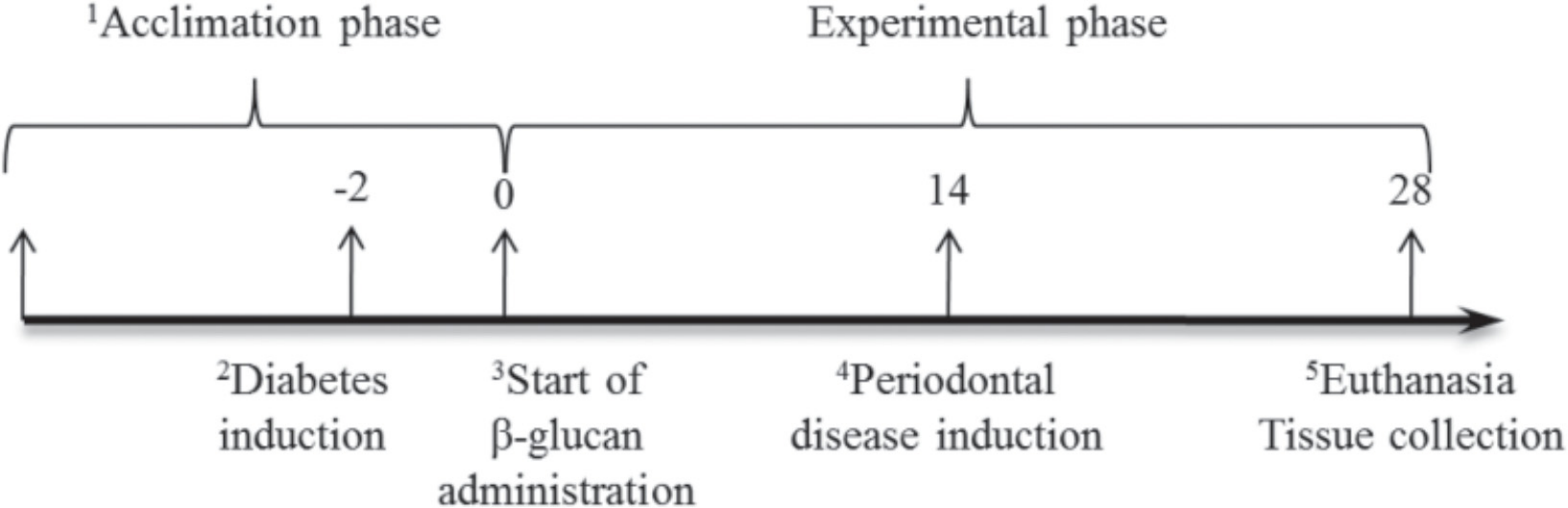
Schematic representation of the experimental design across time. ^1^All animals were acclimated in individual metabolic cages for seven days; ^2^For animals in the following groups: diabetes; diabetes + periodontal disease; diabetes + β-glucan; diabetes + periodontal disease + β-glucan; ^3^For animals in the following groups: β-glucan; diabetes + β-glucan; periodontal disease + β-glucan; diabetes + periodontal disease + β-glucan; ^4^For animals in the following groups: periodontal disease; diabetes + periodontal disease; periodontal disease + β-glucan; diabetes + periodontal disease + β-glucan; ^5^Collection of blood samples and removal of the jaws.

Experimental groups were divided as follows: Group 1—control (without diabetes induction, without periodontal disease induction; without β-glucan administration); Group 2—diabetes control (with diabetes induction; without periodontal disease induction; without β-glucan administration); Group 3—periodontal disease control (with periodontal disease induction; without diabetes induction; without β-glucan administration); Group 4—β-glucan (with β-glucan administration; without diabetes induction; without periodontal disease induction); Group 5—Diabetes and periodontal disease; Group 6—Diabetes with β-glucan administration; Group 7—Periodontal disease with β-glucan administration; Group 8—Diabetes and periodontal disease with β-glucan administration (Fig 1).

### Diabetes induction

To induce experimental diabetes *mellitus*, half of the animals received an intraperitoneal injection with 80 mg/kg of streptozotocin (Sigma, St. Louis, MO, USA) dissolved in citrate buffer [25], administered 48 hours before the experiment started (Fig 1). Animals of the groups without diabetes received only citrate buffer. Before starting the β-glucan administration, the animals were submitted to eight hours of fasting and blood glucose was measured, through amputation of the tail tip, using a glucose meter (Accu-Chek Roche, Basel, Swiss). Animals with a glucose serum level above 200 mg/dL after fasting were considered diabetic, from one reading before the beginning of experiment [26,27].

### Induction of periodontal disease

Periodontal disease was induced in groups 3, 5, 7, and 8 in the first right mandibular molar through a ligature protocol. The animals received general anesthesia through an intraperitoneal injection of xylazine hydrochloride at 10 mg/kg and ketamine hydrochloride at 80 mg/kg. Ligatures were placed under anesthesia to induce periodontal disease [28]. Periodontal disease was induced on the 14^th^ day and ligatures remained in place until the 28^th^ day, when the animals were euthanized (Fig 1).

### β-glucan administration

The β-glucans used in the present study came from *Saccharomyces cerevisiae* leaven and the solution had the following composition: β-glucans—Min. 60.0%; Crude Protein—Max. 8.0%; pH (solution 2%) 4.0–7.0; Ash—Max.10.0g/100g. Distribution of particle size: mean—41 μm; < 20 μm 19%; 20–50 μm 43%; 50–100 μm 28%; 100–200 μm 10%; >200 μm 0%. Fluidity (seconds) – 70.2; Angle of repose (degrees) 31.2; Compressibility 37%; Water retention capacity (mean) 7.4; Solubility rate in water 7.9.

Daily doses of 30 mg/kg/day of β-glucans dissolved in 0.3 ml of saline solution were administered by gavage each morning for 28 days. The non-treated groups of animals received the same volume of saline solution. The animals with periodontal disease received β-glucans during the 14 days before induction and also during the 14 days after ligature placement, amounting to 28 days of treatment with β-glucans (Fig 1).

### Collection of material for analysis

At the end of the 28-day experimental period, blood glucose was measured through tail tip amputation using a glucometer (Accu-Chek Roche, Basel, Swiss) after a fasting period of 8 hours. Afterwards, they were killed through cardiac puncture under anesthesia with intraperitoneal 50 mg/kg thiopental sodium.

Blood samples were collected to determine total cholesterol plasmatic concentrations and triacylglycerol by colorimetric essay using commercial kits (Gold Analisa, Belo Horizonte, Brazil). Plasmatic concentrations of C-Peptide were determined by ELISA assay using commercial kits (Merck Millipore, Solna, Sweden). The analysis were performed in duplicate and all procedures were performed according to kit protocol. The absorbance was read at 450nm in a plate reader (Epoch–BioTek, Winooski, USA).

Immediately after euthanasia, the soft tissues of the jaws (marginal gingiva) were removed with the aid of sterile forceps and bistoury and then were stored in liquid nitrogen for subsequent ribonucleic acid (RNA) extraction. These tissues were used for COX-2 (cyclooxygenase 2), RANK-L (receptor activator of nuclear factor kappa-B ligand) and OPG (osteoprotegerin) gene expression analyses, carried out through quantitative polymerase chain reaction in real time (RT-PCR). The levels of alveolar bone loss were assessed directly in the dissected mandible after removal of soft tissues by means of morphometric analysis [29].

All analyses were blindly performed by trained evaluators.

### RT-PCR for COX-2, RANK-L and OPG genes

Total RNA was isolated using a Qiagen RNeasy Mini Kit (Qiagen; Valencia, CA, USA), according to the manufacturer’s recommendations. Total RNA concentration was determined from optical density using a micro volume spectrophotometer (Nanodrop 1000, Nanodrop Technologies LLC, Wilmington, NC, USA). Total RNA reverse transcription was performed using DNase (Turbo DNA-frees, Ambion Inc., Austin, TX, USA), and one microgram was used for cDNA synthesis. The reaction was performed using the First Strand cDNA synthesis kit (Ferments, Glen Burnie, MD, USA), following the manufacturer’s recommendations.

Primer sets for genes COX2, RANK-L, OPG and glyceraldehyde-3-phosphate dehydrogenase (GAPDH) were designed based on sequences available on GenBank using Primer Express 3.0, a probe design software (Applied Biosystem, Foster City, CA, EUA). Primers sequences were the following: COX2: forward: 5’—ACT TGC GTT GAT GGT GGC TGT CTT—3’, reverse: 5’—CTG TAT CCC GCC CTG CTG GTG—3’; OPG: forward: 5’–TCC TGG CAC CTA CCT AAA ACA GCA—3’, reverse: 5’—CTA CAC TCT CGG CAT TCA CTT TGG—3’; RANKL: forward: 5’–CAT CGG GTT CCC ATA AAG—3’, reverse: 5’–GAA GCA AAT GTT GGC GTA—3’; and GAPDH: forward: 5'–GAC TGT GGA TGG CCC CTC TG-3', reverse: 5'–CGC CTG CTT CAC CAC CTT CT—3'. The RT-PCR was performed using a 7300 PCR Real Time machine (Applied Biosystem) and SYBR Green PCR Master Mix (Ferments, Glen Burnie, MD, USA). The reaction product was quantified with the relative quantification tool, using GAPDH as a reference gene. Negative controls with SYBR Green PCR Master Mix and water were made for all reactions.

### Alveolar bone loss morphometric analysis

Mandibles were dissected and immersed in sodium hypochlorite 1% solution for four hours and the whole remaining bone soft tissue was mechanically removed. The pieces were colored with methylene blue (1g/100ml) for one minute to delimit the cement-enamel junction. Mandibles were observed with a stereoscopic magnifying glass and positioned so that vestibular and lingual cuspids overlapped, with no possibility of viewing the occlusal surface. Digital images were taken using a camera. A previously described method [30] was used to perform linear measurement of the cement-enamel junction at the alveolar bone crest, at half of each root, following the axis. Measurements were obtained of the three roots of first molar to the vestibular face, using the Image J software package (Bethesda, MD, USA). These measurements were obtained by a trained examiner and without knowledge of experimental groups. The average of three measurements per animal was used to express alveolar bone loss.

### Statistical analysis

Statistical analyses were performed using Variance Analysis (ANOVA). When F values indicated significant interactions, these were unfolded between factors. The analyses were performed in the statistical program SISVAR [31] at significance level fixed at 95%.

## Results

A significant difference in mean initial blood glucose was found between the diabetic and non-diabetic groups (p < 0.05). The mean initial blood glucose of animals from the diabetic groups was 428 ± 18 mg/dL.

It was observed that the metabolic parameters criteria, namely analyzed: final blood glucose, total cholesterol and triacylglycerols, were higher in diabetic animal groups compared to non-diabetic groups (Table 1—p < 0.05). Animals with diabetes and periodontal disease have shown higher glucose plasmatic and cholesterol concentrations when compared to diabetic animals without periodontal disease (Table 1—p < 0.05). The triacylglycerols, on the other hand, showed opposite results; animals with diabetes and periodontal disease treated or not with β-glucan have presented lower concentrations against those diabetic with and without β-glucan (Table 1—p < 0.05). β-glucans reduced blood glucose and triacylglycerol levels in diabetic animals, both with and without periodontal disease. When compared to the non-treated group, cholesterol concentrations decreased due to the use of β-glucan in diabetic animals with periodontal disease (Table 1—p < 0.05).

**Table 1:** Glucose, total cholesterol and triacylglycerols levels (mg/dL—mean ± standard deviation) of animals treated with β-glucans from *Saccharomyces cerevisiae* (30mg/kg/day) during 28 days.

DIABETES	PERIODONTAL	β-GLUCANS	
	DISEASE	Without	With
***Glucose***			
Without *	Without	91 (9)	97 (4)
	With	111 (4)	106 (11)
With	Without	517 (32) ^a^ ^x^	367 (24) ^a^ ^y^
	With	561 (44) ^b^ ^x^	440 (73) ^b^ ^y^
***Total cholesterol***			
Without *	Without	50 (5)	53 (4)
	With	59 (5)	58 (9)
With	Without	82 (18) ^a^	72 (10) ^a^
	With	97 (5) ^b^ ^x^	83 (10) ^b^ ^y^
***Triacylglycerols***			
Without *	Without	82 (16)	68 (13)
	With	68 (13)	63 (16)
With	Without	276 (49) ^b^ ^x^	191 (16) ^b^ ^y^
	With	229 (72) ^a^ ^x^	144 (25) ^a^ ^y^

^a,b^ Means followed by different letters in columns indicate significant differences between groups with and without periodontal disease by F test (p < 0,05).

^x,y^ Means followed by different letters in lines indicate significant difference between groups with and without β-glucans ingestion by F test (p < 0,05).

* Significant difference between groups with and without diabetes by F test (p < 0,05)

In general, animals with periodontal disease presented lower levels of C-peptide than those without the disease, except animals with diabetes treated with β-glucans (Table 2—p < 0.05). In animals not receiving β-glucans, C-peptide levels were lower in diabetic animals when compared to non-diabetic ones (Table 2—p < 0.05). C-peptide levels in diabetic animals with periodontal disease treated with β-glucans were higher than the untreated (Table 2—p < 0.05).

**Table 2:** Serum levels of C-peptide (pM—mean ± standard deviation) of animals treated with β-glucans from *Saccharomyces cerevisiae* (30mg/kg/day) during 28 days.

DIABETES	PERIODONTAL	β-GLUCANS	
	DISEASE	Without	With
Without	Without	413 (56) ^b^ ^B^	395 (73) ^b^ ^B^
	With	277 (108) ^a^ ^B^	310 (51) ^a^
With	Without	226 (14) ^b^ ^A^	252 (41) ^A^
	With	119 (2) ^a^ ^A^ ^y^	280 (8) ^x^

^A,B^ Means followed by different letters in columns indicate significant differences between groups with and without diabetes by F test (p < 0,05).

^a,b^ Means followed by different letters in columns indicate significant differences between groups with and without periodontal disease by F test (p < 0,05).

^x,y^ Means followed by different letters in lines indicate significant differences between groups with and without β-glucan ingestion by F test (p < 0,05).

**Fig 2:**
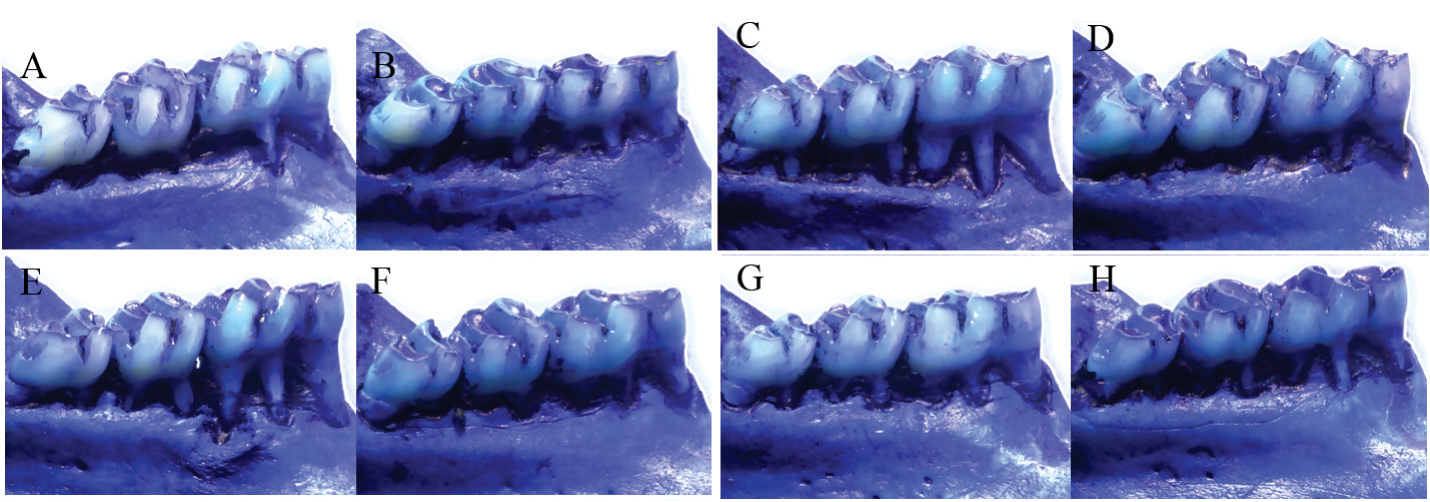
Alveolar bone loss in animals treated with β-glucans (30 mg/kg/day for 28 days). A—control; B—diabetes; C—periodontal disease; D—β-glucan; E—diabetes + periodontal disease; F—diabetes + β-glucan; G—periodontal disease + β-glucan, H—diabetes + periodontal disease + β-glucan. Only one representative animal per group is depicted in this figure.

The groups of animals with periodontal disease presented higher alveolar bone loss than the groups without the disease induction (Fig 2 and Table 3—p < 0.05). Diabetic animals with periodontal disease exhibited more alveolar bone loss than the animals with diabetes and without periodontal disease (Table 3—p < 0.05). The administration of β-glucans has reduced alveolar bone loss in both diabetic and non-diabetic animals with periodontal disease (Table 3—p < 0.05).

**Table 3:** Alveolar bone loss (mm—mean ± standard deviation) in animals treated with β-glucans from *Saccharomyces cerevisiae* (30mg/kg/day) during 28 days.

DIABETES	PERIODONTAL	β-GLUCANS	
	DISEASE	Without	With
Without	Without	1,20 (0,14) ^a^	0,98 (0,12) ^a^
	With	2,10 (0,36) ^b^ ^A^ ^x^	1,78 (0,35) ^b^ ^A^ ^y^
With	Without	1,16 (0,10) ^a^	1,13 (0,13) ^a^
	With	2,63 (0,13) ^b^ ^B^ ^x^	2,10 (0,36) ^b^ ^B^ ^y^

^A,B^ Means followed by different letters in columns indicate significant differences between groups with and without diabetes by F test (p < 0,05).

^a,b^ Means followed by different letters in columns indicate significant differences between groups with and without periodontal disease by F test (p < 0,05).

^x,y^ Means followed by different letters in lines indicate significant differences between groups with and without β-glucan ingestion by F test (p < 0,05).

The Fig 2 showed, illustratively, the progression of periodontal disease (indicated by alveolar bone loss parameter) in animals which were submitted to the ligation protocol (Fig 2C). It was observed a aggravation of the disease when they were diabetic (Fig 2E). In all the groups which received treatment with β-glucan was observed a decrease in alveolar bone loss (Fig 2F, 2G and 2H) when they were compared to the respective untreated groups (Fig 2B, 2C and 2E). Again, this figure is simply illustrative. The results regarding to alveolar bone loss are presented in Table 3In diabetic animals, the presence of periodontal disease has caused an increase in the expression of COX-2 (Table 4—p < 0.05). The treatment with β-glucans has reduced the expression of this gene in animals with periodontal disease, both diabetics and non-diabetics, against those that were not treated (Table 4—p < 0.05).

**Table 4:** COX-2, RANK-L and OPG gene expression (mean ± standard deviation) of animals treated with β-glucan from *Saccharomyces cerevisiae* (30mg/kg/day) during 28 days.

DIABETES	PERIODONTAL	β-GLUCANS	
	DISEASE	Without	With
***COX-2***			
Without	Without	1,08 (0,18)	0,89 (0,12)
	With	1,40 (0,44) ^x^	0,92 (0,24) ^y^
With	Without	0,84 (0,07) ^a^	0,72 (0,15)
	With	1,54 (0,44) ^b^ ^x^	0,82 (0,08) ^y^
***RANK-L***			
Without	Without	1,46 (0,42) ^a^	1,54 (0,53) ^A^
	With	4,21 (1,60) ^b^ ^A^ ^x^	2,41 (0,63) ^y^
With	Without	2,22, (0,32) ^a^	3,71 (0,93) ^B^
	With	6,74(2,46) ^b^ ^B^ ^x^	2,09 (0,65) ^y^
***OPG***			
Without	Without	3,86 (0,52) ^b^ ^B^	3,92 (0,82) ^b^ ^B^
	With	1,17 (0,33) ^a^ ^y^	2,57 (0,21) ^a^ ^x^
With	Without	2,24 (0,33) ^A^	2,12 (0,96) ^A^
	With	1,47 (0,36)	2,14 (0,63)

^A,B^ Means followed by different letters in columns indicate significant differences between groups with and without diabetes by F test (p < 0,05).

^a,b^ Means followed by different letters in columns indicate significant differences between groups with and without periodontal disease by F test (p < 0,05).

^x,y^ Means followed by different letters in lines indicate significant differences between groups with and without β-glucan ingestion by F test (p < 0,05).

Animals with diabetes and periodontal disease showed higher expression of RANK-L compared to non-diabetic animals with periodontal disease (Table 4—p < 0.05). A reduction in the expression of this gene was observed in animals with periodontal disease and diabetes in function of β-glucan treatment (Table 4—p < 0.05). In untreated animals, the groups with periodontal disease showed higher expression of RANK-L (Table 4—p < 0.05).

With regard to OPG expression in non-diabetic animals, groups without periodontal disease have presented higher OPG expression than the groups with periodontal disease (Table 4—p < 0.05). These same groups (non-diabetic and without periodontal disease) have also presented a higher OPG expression when compared to diabetic animals without periodontal disease (Table 4—p < 0.05). Treatment with β-glucans resulted in increased OPG expression in non-diabetic animals with periodontal disease (Table 4—p< 0.05).

No animals were lost during the experimental period. All animals were included in the statistical analyses (6/6 per group). At the end of the experiment, the animals presented good health status.

## Discussion

The results of the present study demonstrate that β-glucans were efficient in improving metabolic parameters (blood glucose, total cholesterol and triacylglycerols) and reducing alveolar bone loss in diabetic animals with periodontal disease.

The positive effects of β-glucans on the metabolism of diabetic rats are known [21,32]. The improvement of these parameters with the use of β-glucans may be associated to the ability of these compounds to act directly in digestion. These polysaccharides form a gelatinous layer that works as a barrier, that renders carbohydrate absorption difficult and thus leading to lower concentrations of glucose and lipids in the blood [22,23]. Our results are in agreement with a previous study that showed significant improvement in blood glucose, triacylglycerols and total cholesterol in diabetic rats treated with β-glucans, elucidating its beneficial effect on glucose tolerance and lipid metabolism [33,34].

Animals with periodontal disease presented lower levels of C-peptide. This result may be associated with high cytokine levels (interleukin-1 beta, tumor necrosis factor alpha), which increase insulin resistance and induce pancreatic beta cell apoptosis [35]. C-peptide levels in diabetic animals with periodontal disease treated with β-glucans were increased in relation to animals in the same condition that did not received β-glucans. The effects of β-glucans in the modulation of inflammatory profile (as observed in COX-2 expression) can indicate a reduction in the gingival inflammation and a preventive effect was observed in beta cell C-peptide secretion [36].

The worsening of alveolar bone loss observed in diabetic animals with periodontal disease corroborates previous studies, since there is a bidirectional relationship between these diseases [12,20,37]. However, blood glucose control seemed not to be the only factor associated with the action of β-glucans in preventing alveolar bone loss, since it was also effective in rats with normal glucose levels. Such result may be associated with the immunomodulatory properties of β-glucans, specifically the reduction of RANK-L expression (a parameter associated with alveolar bone loss). Thus, we can associate the improvement in alveolar bone loss (both in diabetic and non-diabetic animals) to the known immunomodulatory effects of β-glucans from fungi [19,38,39]. The immune action of these polysaccharides is associated with their capacity to activate leucocytes, stimulating their phagocytic and cytotoxic functions and antimicrobial activity [40,41].

In the present study, animals with induced periodontal disease exhibited high levels of RANK-L and reduction in OPG expression. The ratio RANK-L/OPG balance is essential to bone tissue homeostasis [42]. OPG regulates bone remodeling through controlling osteoclast differentiation and activation [43,44]. On the other hand, RANK-L favors osteoclastogenesis, stimulating osteoclastic activity and inhibiting apoptosis [44,45]. RANK-L and OPG involvement in periodontal disease was evidenced in a study where mice were induced to periodontitis through inoculation of a pathogenic micro-organism. In this model, the periodontal disease activity was associated with high levels of RANK-L, while the increase in OPG expression inhibited this binding action [46]. Similar results to those of the present study were also found in experiments with diabetic mice induced by inoculation of bacteria, which showed higher RANK-L expression in sick animals. These results indicated that there was less bone loss when higher levels of OPG were observed [47]. Similar results were found in studies with rats induced to periodontal disease by ligature, where OPG was associated with alveolar bone volume preservation [48].

In the present study, the use of β-glucans has reduced RANK-L expression in animals with periodontal disease, while an increase in OPG expression was observed in non-diabetic animals with periodontal disease. Once again this result may indicate a modulating effect of β-glucans on the immune system [33,49]. Similarly, the lower COX-2 expression in diabetic animals with periodontal disease treated with β-glucans may be attributed to the anti-inflammatory activity of these compounds.

The hypothesis of this study was to investigate the effects of β-glucans in concomitant periodontal disease and diabetes mellitus. The results presented in this study showed that treatment with β-glucans improved blood glucose control and attenuated alveolar bone loss.

The present study has demonstrated that β-glucans from *Saccharomyces cerevisiae* present both immunomodulatory and metabolic properties, and may be beneficial for diabetic patients with periodontal disease. There are no reports of toxicity associated with β-glucan consumption, which makes this compound relatively safe for consumption [15]. Hence, β-glucans arise as a feasible complementary treatment for those patients in the future.

Considering that alveolar bone loss is also prevalent in patients with diabetes *mellitus* type 2, it would be interesting to evaluate the effects of β-glucans in models of type 2 diabetes associated with induced periodontal disease.

## Conclusion

Treatment with β-glucans from *Saccharomyces cerevisiae* administered during a 28-day period reduced blood glucose levels and attenuated alveolar bone loss in diabetic rats with periodontal disease.

## Supporting Information

S1 Checklist The ARRIVE Guidelines Checklist Animal Research: Reporting In Vivo Experiments. (PDF)

S1 Fig Schematic representation of the experimental design across time. 1All animals were acclimated in individual metabolic cages for seven days; 2For animals in the following groups: diabetes; diabetes + periodontal disease; diabetes + β-glucan; diabetes + periodontal disease + β-glucan; 3For animals in the following groups: β-glucan; diabetes + β-glucan; periodontal disease + β-glucan; diabetes + periodontal disease + β-glucan; 4For animals in the following groups: periodontal disease; diabetes + periodontal disease; periodontal disease + β-glucan; diabetes + periodontal disease + β-glucan; 5Collection of blood samples and removal of the jaws.
(TIFF)

S2 Fig Alveolar bone loss in animals treated with β-glucans (30 mg/kg/day for 28 days). A—control; B—diabetes; C—periodontal disease; D—β-glucan; E—diabetes + periodontal disease; F—diabetes + β-glucan; G—periodontal disease + β-glucan, H—diabetes + periodontal disease + β-glucan. Only one representative animal per group is depicted in this figure.
(TIFF)

S1 Table Glucose, total cholesterol and triacylglycerols levels (mg/dL—mean β standard deviation) of animals treated with β-glucans from Saccharomyces cerevisiae (30mg/kg/day) during 28 days. (DOCX)

S2 Table Serum levels of C-peptide (pM—mean β standard deviation) of animals treated with β-glucans from Saccharomyces cerevisiae (30mg/kg/day) during 28 days. (DOCX)

S3 Table Alveolar bone loss (mm—mean ± standard deviation) in animals treated with β-glucans from Saccharomyces cerevisiae (30mg/kg/day) during 28 days. (DOCX)

S4 Table COX-2, RANK-L and OPG gene expression (mean ± standard deviation) of animals treated with β-glucan from Saccharomyces cerevisiae (30mg/kg/day) during 28 days. (DOCX)
