# Supplementary figures and images for: β-Glucans (Saccharomyces cereviseae) Reduce Glucose Levels and Attenuate Alveolar Bone Loss in Diabetic Rats with Periodontal Disease
Source: PLoS One. 2015 Aug 20;10(8):e0134742. doi: 10.1371/journal.pone.0134742 (PMC4546386; doi:10.1371/journal.pone.0134742)

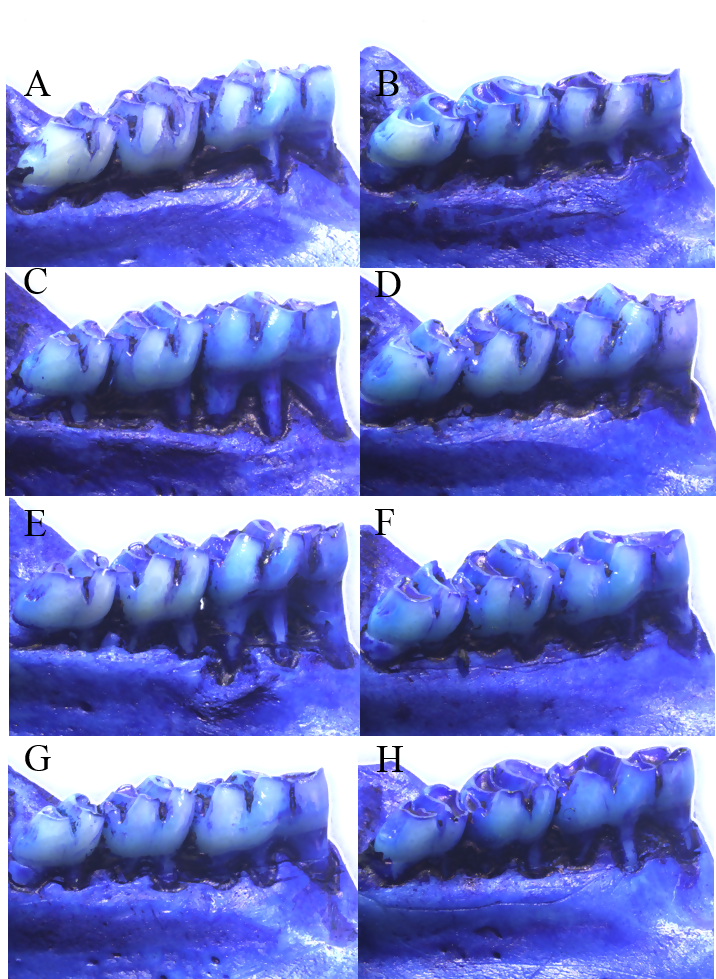

Supplement: S2 Fig — A—control; B—diabetes; C—periodontal disease; D—β-glucan; E—diabetes + periodontal disease; F—diabetes + β-glucan; G—periodontal disease + β-glucan, H—diabetes + periodontal disease + β-glucan. Only one representative animal per group is depicted in this figure. (TIFF) [file pone.0134742.s003.tiff]
