# Supplementary material for: β-Glucans (Saccharomyces cereviseae) Reduce Glucose Levels and Attenuate Alveolar Bone Loss in Diabetic Rats with Periodontal Disease
Source: PLoS One. 2015 Aug 20;10(8):e0134742. doi: 10.1371/journal.pone.0134742 (PMC4546386; doi:10.1371/journal.pone.0134742)
Supplement: S1 Table — (DOCX) [file pone.0134742.s004.docx]

**S1 Table:** Glucose, total cholesterol and triacylglycerols levels (mg/dL - mean ± standard deviation) of animals treated with β-glucans from *Saccharomyces cerevisiae* (30mg/kg/day) during 28 days

| DIABETES | PERIODONTAL DISEASE | | β-GLUCANS | |
| --- | --- | --- | --- | --- |
|  |  |  | Without | With |
| ***Glucose*** | | | | |
| Without * | | Without | 91 (9) | 97 (4) |
|  |  | With | 111 (4) | 106 (11) |
| With | | Without | 517 (32) ^a x^ | 367 (24) ^a y^ |
|  |  | With | 561 (44) ^b x^ | 440 (73) ^b y^ |
| ***Total cholesterol*** | | | | |
| Without * | | Without | 50 (5) | 53 (4) |
|  |  | With | 59 (5) | 58 (9) |
| With | | Without | 82 (18) ^a^ | 72 (10) ^a^ |
|  |  | With | 97 (5) ^b x^ | 83 (10) ^b y^ |
| ***Triacylglycerols*** | | | | |
| Without * | | Without | 82 (16) | 68 (13) |
|  |  | With | 68 (13) | 63 (16) |
| With | | Without | 276 (49) ^b x^ | 191 (16) ^b y^ |
|  |  | With | 229 (72) ^a x^ | 144 (25) ^a y^ |

* Significant difference between groups with and without diabetes by F test (p < 0,05)

^a,b^ Means followed by different letters in columns indicate significant differences between groups with and without periodontal disease by F test (p < 0,05)

^x,y^ Means followed by different letters in lines indicate significant difference between groups with and without β-glucans ingestion by F test (p < 0,05)
