# Supplementary material for: β-Glucans (Saccharomyces cereviseae) Reduce Glucose Levels and Attenuate Alveolar Bone Loss in Diabetic Rats with Periodontal Disease
Source: PLoS One. 2015 Aug 20;10(8):e0134742. doi: 10.1371/journal.pone.0134742 (PMC4546386; doi:10.1371/journal.pone.0134742)
Supplement: S2 Table — (DOCX) [file pone.0134742.s005.docx]

**S2 Table:** Serum levels of C-peptide (pM - mean ± standard deviation) of animals treated with β-glucans from *Saccharomyces cerevisiae* (30mg/kg/day) during 28 days

| DIABETES | PERIODONTAL DISEASE | β-GLUCANS | |
| --- | --- | --- | --- |
|  |  | Without | With |
| Without | Without | 413 (56) ^b B^ | 395 (73) ^b B^ |
|  | With | 277 (108) ^a B^ | 310 (51) ^a^ |
| With | Without | 226 (14) ^b A^ | 252 (41) ^A^ |
|  | With | 119 (2) ^a A y^ | 280 (8) ^x^ |

^A,B^ Means followed by different letters in columns indicate significant differences between groups with and without diabetes by F test (p < 0,05)

^a,b^ Means followed by different letters in columns indicate significant differences between groups with and without periodontal disease by F test (p < 0,05)

^x,y^ Means followed by different letters in lines indicate significant differences between groups with and without β-glucan ingestion by F test (p < 0,05)
