# Supplementary material for: β-Glucans (Saccharomyces cereviseae) Reduce Glucose Levels and Attenuate Alveolar Bone Loss in Diabetic Rats with Periodontal Disease
Source: PLoS One. 2015 Aug 20;10(8):e0134742. doi: 10.1371/journal.pone.0134742 (PMC4546386; doi:10.1371/journal.pone.0134742)
Supplement: S3 Table — (DOCX) [file pone.0134742.s006.docx]

**S3 Table:** Alveolar bone loss (mm - mean ± standard deviation) in animals treated with β-glucans from *Saccharomyces cerevisiae* (30mg/kg/day) during 28 days

| DIABETES | PERIODONTAL DISEASE | β-GLUCANS | |
| --- | --- | --- | --- |
|  |  | Without | With |
| Without | Without | 1,20 (0,14) ^a^ | 0,98 (0,12) ^a^ |
|  | With | 2,10 (0,36) ^b A x^ | 1,78 (0,35) ^b A y^ |
| With | Without | 1,16 (0,10) ^a^ | 1,13 (0,13) ^a^ |
|  | With | 2,63 (0,13) ^b B x^ | 2,10 (0,36) ^b B y^ |

^A,B^ Means followed by different letters in columns indicate significant differences between groups with and without diabetes by F test (p < 0,05)

^a,b^ Means followed by different letters in columns indicate significant differences between groups with and without periodontal disease by F test (p < 0,05)

^x,y^ Means followed by different letters in lines indicate significant differences between groups with and without β-glucan ingestion by F test (p < 0,05)
