# Supplementary material for: β-Glucans (Saccharomyces cereviseae) Reduce Glucose Levels and Attenuate Alveolar Bone Loss in Diabetic Rats with Periodontal Disease
Source: PLoS One. 2015 Aug 20;10(8):e0134742. doi: 10.1371/journal.pone.0134742 (PMC4546386; doi:10.1371/journal.pone.0134742)
Supplement: S4 Table — (DOCX) [file pone.0134742.s007.docx]

**S4 Table:** COX-2, RANK-L and OPG gene expression (mean ± standard deviation) of animals treated with β-glucan from *Saccharomyces cerevisiae* (30mg/kg/day) during 28 days

| DIABETES | PERIODONTAL DISEASE | | β-GLUCANS | |
| --- | --- | --- | --- | --- |
|  |  |  | Without | With |
| ***COX-2*** | | | | |
| Without | | Without | 1,08 (0,18) | 0,89 (0,12) |
|  |  | With | 1,40 (0,44) ^x^ | 0,92 (0,24) ^y^ |
| With | | Without | 0,84 (0,07) ^a^ | 0,72 (0,15) |
|  |  | With | 1,54 (0,44) ^b x^ | 0,82 (0,08) ^y^ |
| ***RANK-L*** | | | | |
| Without | | Without | 1,46 (0,42) ^a^ | 1,54 (0,53) ^A^ |
|  |  | With | 4,21 (1,60) ^b A x^ | 2,41 (0,63) ^y^ |
| With | | Without | 2,22, (0,32) ^a^ | 3,71 (0,93) ^B^ |
|  |  | With | 6,74(2,46) ^b B x^ | 2,09 (0,65) ^y^ |
| ***OPG*** | | | | |
| Without | | Without | 3,86 (0,52) ^b B^ | 3,92 (0,82) ^b B^ |
|  |  | With | 1,17 (0,33) ^a y^ | 2,57 (0,21) ^a x^ |
| With | | Without | 2,24 (0,33) ^A^ | 2,12 (0,96) ^A^ |
|  |  | With | 1,47 (0,36) | 2,14 (0,63) |

^A,B^ Means followed by different letters in columns indicate significant differences between groups with and without diabetes by F test (p < 0,05)

^a,b^ Means followed by different letters in columns indicate significant differences between groups with and without periodontal disease by F test (p < 0,05)

^x,y^ Means followed by different letters in lines indicate significant differences between groups with and without β-glucan ingestion by F test (p < 0,05)
